# Application of Biodegradable and Biocompatible Nanocomposites in Electronics: Current Status and Future Directions

**DOI:** 10.3390/nano9070950

**Published:** 2019-06-29

**Authors:** Haichao Liu, Ranran Jian, Hongbo Chen, Xiaolong Tian, Changlong Sun, Jing Zhu, Zhaogang Yang, Jingyao Sun, Chuansheng Wang

**Affiliations:** 1 Academic Division of Engineering, Qingdao University of Science & Technology, Qingdao 266061, China; 2 College of Mechanical and Electrical Engineering, Beijing University of Chemical Technology, Beijing 100029, China; 3 College of Electromechanical Engineering, Qingdao University of Science & Technology, Qingdao 266061, China; 4 College of Sino-German Science and Technology, Qingdao University of Science & Technology, Qingdao 266061, China; 5 College of Pharmacy, The Ohio State University, Columbus, OH 43210, USA; 6 Department of Radiation Oncology, The University of Texas Southwestern Medical Center, Dallas, TX 75390, USA

**Keywords:** biodegradable, biocompatible, electronics, nanocomposites

## Abstract

With the continuous increase in the production of electronic devices, large amounts of electronic waste (E-waste) are routinely being discarded into the environment. This causes serious environmental and ecological problems because of the non-degradable polymers, released hazardous chemicals, and toxic heavy metals. The appearance of biodegradable polymers, which can be degraded or dissolved into the surrounding environment with no pollution, is promising for effectively relieving the environmental burden. Additionally, biodegradable polymers are usually biocompatible, which enables electronics to be used in implantable biomedical applications. However, for some specific application requirements, such as flexibility, electric conductivity, dielectric property, gas and water vapor barrier, most biodegradable polymers are inadequate. Recent research has focused on the preparation of nanocomposites by incorporating nanofillers into biopolymers, so as to endow them with functional characteristics, while simultaneously maintaining effective biodegradability and biocompatibility. As such, bionanocomposites have broad application prospects in electronic devices. In this paper, emergent biodegradable and biocompatible polymers used as insulators or (semi)conductors are first reviewed, followed by biodegradable and biocompatible nanocomposites applied in electronics as substrates, (semi)conductors and dielectrics, as well as electronic packaging, which is highlighted with specific examples. To finish, future directions of the biodegradable and biocompatible nanocomposites, as well as the challenges, that must be overcome are discussed.

## 1. Introduction

Electronic products have enhanced our lives and brought about changes in almost all areas, including communications, manufacturing, entertainment, and health care [1]. With the rapid renewal of electronic products, such as smartphones and tablets, the life of electronic products is becoming shorter. As a result, an increasing amount of electronic waste (E-waste) is routinely discarded [2,3]. The fastest growing type of E-waste is solid waste. Not only is solid E-waste comprised of a large amount of non-degradable polymers, but it also releases hazardous chemicals and toxic heavy metals, both of which are damaging to the environment and ecology [4,5]. For certain electronic products, this damage would start with raw material procurement and continue throughout the whole life cycle [2].

Biodegradable electronics may be an effective solution for E-waste management, since they can be degraded or dissolved into the surrounding environment with no pollution. This endows the electronics with environmental safety and disposability [6,7,8], by simultaneously decreasing the cost for recycling operations and the health risks associated with harmful emissions [9,10,11,12].

Additionally, biodegradable materials are usually biocompatible, which enables electronics to be used in implantable biomedical applications. Biocompatibility allows the materials to directly contact tissues or skin without generating adverse effects [13,14,15,16,17]. Furthermore, electronics which are both biodegradable and biocompatible can be dissolved or resorbed safely by human body at controlled rates after treatment or diagnosis is completed. Eliminating the need for a second surgery to retrieve the device simultaneously decreases the associated infection risks [18].

Besides biodegradability and biocompatibility, some other characteristics, including flexibility, mechanical properties, electric conductivity, and gas and vapor barrier properties, are also essential for specific applications in electronics. However, many polymers cannot completely meet these performance requirements. Therefore, recent research has focused on incorporating nanofillers with excellent properties into polymers so as to improve their performance capabilities [19,20,21,22].

This paper aims to carefully demonstrate the development and potential of the biodegradable and biocompatible nanocomposites in electronic applications. It will first review emergent biodegradable and biocompatible polymers used as insulators or (semi)conductors, and then highlight specific examples of nanocomposites used in electronics as substrates, conductors, semiconductors, and dielectrics, as well as electronic packaging [23].

## 2. Biodegradable and Biocompatible Polymers

Biopolymers are the basis of biodegradable and biocompatible nanocomposites. They can be classified as natural-based polymers and synthetic polymers [13]. Natural-based polymers refer to those which come from nature. Table 1 shows an overview of biodegradable and biocompatible polymers used to fabricate electronics. In this section, biodegradable and biocompatible polymers will be introduced according to their conductivity, since the electrical property directly determines their application directions.

### 2.1. Insulated Polymers

**Figure 1:**
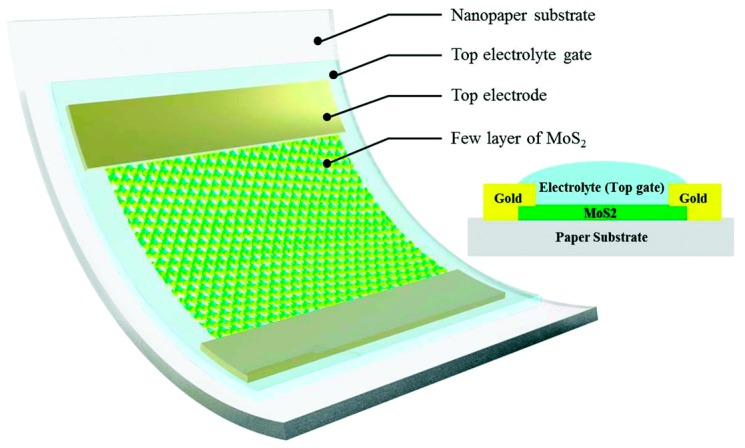
Three-dimensional schematic and cross-sectional view of the MoS_2_ phototransistor, fabricated on flexible and transparent cellulose nanopaper. Reproduced with permission from [61], RSC, 2016.

Cellulose, as a macromolecule polysaccharide composed of glucose, is the oldest and cheapest biodegradable natural source polymer. It is inexpensive, biodegradable, abundant, easily available, and lightweight, and thus is considered to be a potential substitute for the substrate materials of various electronic devices, including organic field-effect transistors (OFETs), organic light-emitting diodes (OLEDs), and solar cells [56,57,58,59,60]. For example, Zhang et al. [61] introduced a MoS_2_ phototransistor with a flexible and transparent paper substrate (fabricated from cellulose), as shown in Figure 1. The phototransistor has a high transparency with an average transmittance of 82%. Aside from its use as a substrate, cellulose can also be used to fabricate dielectrics [26,62,63]. Dai et al. [64] fabricated a class of all solid-state ionic dielectrics using cellulose nanopaper. These dielectrics show high transparency, low surface roughness, good thermal durability, and excellent mechanical properties. The successful applications of cellulose as substrates and dielectric materials demonstrate its potential for use in flexible, environmentally friendly and biodegradable electronic devices.

Silk is a polypeptide polymer mainly composed of fibroin and sericin [65]. Because of its outstanding mechanical properties, flexibility, processability, and chemical stability, silk is an ideal backbone for flexible and stretchable electronics [66]. Moreover, silk is non-toxic, completely biodegradable and bioresorbable. It can also be safely implanted into the human body with no immune response, which allows it to be used for implantable electronic therapeutic devices. Kim et al. [67] successfully fabricated an ultrathin electronic sensor array on silk, and tested its performance in vivo by placing it onto exposed brain tissue. The silk was safely dissolved and resorbed, forming a conformal coating on folded brain tissue with the sensor array. Other studies have also demonstrated the successful application of silk as a substrate in implantable electronics [28,68] and food sensors [69]. Applications of silk in dielectrics were also reported [29,70,71,72,73,74,75,76,77]. Liang et al. [70] fabricated organic thin-film transistors (OTFTs) with silk as their dielectric layer. The silk dielectric layer annealed at 40 °C, and had the smallest particles and least aggregation. The mobility of the OTFTs was 2.06 × 10^−3^ cm^2^ V^−1^ s^−1^, and the highest on/off ratio was 10^3^.

Shellac is a natural resin collected from the secretion of the female lac bug after they ingest the sap of their host trees. Shellac can not only be extracted through a variety of polar organic solutions, but it can also be synthesized from a multitude of compositional grades and shades [78,79]. Similar to the aforementioned biopolymers, shellac is biodegradable and can be used as an electronic substrate and a dielectric [30]. Irimia-Vladu et al. [80] reported an organic thin-film transistor (OTFT), which is built on a smooth and uniform shellac film substrate prepared by drop-casting. The OTFT exhibits a mobility of 10^−2^ cm^2^ V^−1^ s^−1^, partially attributed to the outstanding barrier and insulation properties of the shellac film substrate. In addition, shellac also shows excellent dielectric properties. Baek et al. [31] fabricated semiconducting copolymer-based OFETs with shellac and poly(4-vinylphenol) (PVP) as the dielectric materials. The shellac dielectric layer facilitated electron transport at the interface with copolymer channels, endowing the OFETs with superior performances.

Gelatin is another protein-based material, derived from the degradation of collagen in connective tissues, such as animal skin, bone, sarcolemma, and muscle. It is fully biocompatible and biodegradable and most commonly used for oral drug capsules [81]. Nowadays, gelatin is also the basis of many substrates and dielectrics of high-performance electronics [32,33,34,35]. Electronics mounted on hard gelatin substrates can be easily ingested for specific biomedical applications. When used as the gate dielectric in oxide FETs, gelatin could yield a specific capacitance over 0.93 μF cm^−2^ as a result of the formation of electric-double-layers [35].

In addition to natural-based polymers, some synthetic polymers also possess excellent biodegradability and biocompatibility. Poly(vinyl alcohol) (PVA) is one such synthetic polymer and has been widely used in the substrates and dielectrics of electronics [37,38,82,83]. Kim et al. [84] reported an integrated device on the surface of a thin polydimethylsiloxane (PDMS) foil with a water-soluble PVA substrate designed to measure the electrical signals produced by human body. The integrated device includes a set of multifunctional sensors, transistors, capacitors, photo-detectors, oscillators, light-emitting diodes, radio-frequency inductors, and wireless power transmitter coils [85,86,87,88,89]. After the integrated device is mounted on skin, the PVA substrate can easily be washed away. The device also can be peeled off. Furthermore, Afsharimani and Nysten [39] prepared PVA thin films by spin-coating and utilized them as polymer gate dielectrics to fabricate transistors. The transistors show ambipolar behavior with hole and electron mobilities in a low voltage range, indicating a promising potential future in dielectrics.

PDMS is a transparent elastic polymer with excellent biocompatibility [90,91,92]. It has been approved by the US National Heart, Lung, and Blood Institute to be a discriminatory tool for validating the evaluation of biomaterials [38]. Because of its elasticity and biocompatibility, PDMS has been widely used in flexible electronics, and it shows great potential in implantable electronics [93,94,95,96,97]. Delivopoulos et al. [41] developed an implantable monitoring device to record and distinguish two types of bladder afferent activity. The device could survive under immersion in warm saline for three months, exhibiting excellent stability. Disappointingly, PDMS cannot biodegrade easily, greatly limiting its applications.

In addition to the abovementioned polymers, there are some other insulated biodegradable or biocompatible polymers that can be used in electronics, such as starch [98,99], chitosan [100,101], albumen [102], and poly (glycerol-co-sebacate) (PGS) [103], which will not be introduced in detail.

Generally, almost all the insulated biopolymers can be used to fabricate both substrates and dielectrics. When used as substrates, biopolymers should be flexible, lightweight, and processable. However, incorporating nanofillers into substrate materials would seriously decrease the flexibility of the substrate, which is the developing direction for stretchable electronics. Thus, nanofillers are not usually added.

When used as dielectrics, biopolymers must exhibit a significant dielectric property. However, biopolymers cannot always satisfy the requirements of a standard dielectric layer in electronics. Adding certain nanofillers into the biopolymer matrix would significantly improve the dielectric properties. This application will be carefully reviewed in the following section.

### 2.2. Conductive and Semiconductive Polymers

The active materials in electronic devices are usually semiconducting to achieve a certain degree of controllable conductivity, which is the basic principle of most electronics. A conductive or semiconductive polymer is a kind of polymer material with a conjugated π-bond, which can change the polymer from an insulator to a conductor by chemical or electrochemical doping. The basis of the electrical conductivity in conjugated polymers is the delocalization of electrons along the polymer backbone, through the overlap of π-orbitals as well as π-π stacking between polymer chains. Compared with inorganic (semi)conductors, the main advantages of conjugated polymers are mechanical flexibility and lower cost in processing, which allows for inexpensive manufacturing [104]. Many highly conjugated polymers have been developed and applied for (semi)conductive components in various electronics [105].

In addition to electronic conductivity, ionic conductivity also exists in certain polymers, such as melanin and chitosan. Ionic-conducting materials, extensively researched for fuel cell applications, have recently been recognized as having great potential in biocompatible electronics. Additionally, many conducting polymers which can conduct both ionic and electronic currents are extremely well suited to be bioelectronic interface materials. A demonstration of a proton-conducting chitosan thin-film transistor device controlled by the electronic field effect of a gate is a functional realization of the electronic/protonic interface [106].

Melanin is a bio-pigment in animals, plants, and protozoa, formed by a series of chemical reactions of tyrosine or 3,4-dihydroxyphenylalanine. It is a biodegradable and biocompatible natural polymer exhibiting charge transport properties [107,108,109,110], which are believed to possess a mixed protonic/electronic property. The protonic/electronic property is influenced by redox reactions, which can be manipulated by changing the hydration state of the material [111]. When prepared into films, the conductivity of melanin reaches the order of 10^−8^ S cm^−1^ in a dehydrated state, and up to 10^−3^ S cm^−1^ in a fully hydrated state. Bettinger et al. [107] demonstrated a tissue engineering application with melanin as the biodegradable semiconducting material. The melanin film in its fully hydrated state possesses a conductivity of 7 × 10^−5^ S cm^−1^. The fabricated melanin implant exhibits a similar inflammatory response compared with the silicone implant, and it can be completely degraded in vivo after eight weeks, which makes it more promising.

At present, while the application of (semi)conductive natural polymers is still limited, that of synthetic (semi)conductive polymers is relatively mature, such as polyaniline (PANI), polypyrrole (PPy), poly(3,4-ethylenedioxythiophene) (PEDOT). These conjugated polymers exhibit good biocompatibility in biological applications, but their biodegradability is relatively poor. One strategy to combat this is to blend conjugated polymers with biodegradable, insulating polymers to fabricate partially biodegradable composites. The relative composition can be varied to maximize electric conductivity and minimize the proportion of the non-degradable conjugated component.

PANI has attracted great attention because of its high electric conductivity. Beyond that it also has other beneficial characteristics, including facile synthesis, excellent thermal and environmental stability, controllable electric conductivity, appealing electrochemical properties, and reversible doping/dedoping characteristics [112]. PANI has promising future applications in flexible electronics, such as elastic electrodes and strain-sensors [113,114,115]. For example, Huang et al. [116] developed a smart pH self-adjusting switching system using a layer-structured silver nanowire/PANI nanocomposite film, which was fabricated via an easy vertical spinning method. The as-prepared nanocomposite film shows a high electric conductivity of 1.03 × 10^4^ S cm^−1^ at the silver nanowire areal density of 0.84 mg cm^−2^. In addition to electric conductivity, PANI also shows good biocompatibility to cells and tissues, which has been demonstrated in vitro [53] and in vivo [117].

PPy is among the first-studied conductive polymers and has been used widely in bioelectronics and biosensors. It is usually prepared by the oxidation of pyrrole, which can be achieved using ferric chloride or electrochemical polymerization. In the oxidation process, the conductivity of PPy can be greatly affected by the conditions and reagents because dopants could offer additional properties. For example, introducing poly(glutamic acid) as a dopant into PPy would provide pendant carboxylic acid groups, which would further improve electrical conductivity [118]. Similar to PANI, PPy also shows good biocompatibility both in vitro and in vivo [119], but suffers from poor biodegradability.

PEDOT is a conductive polymer based on 3,4-ethylenedioxythiophene (EDOT) monomer. It is produced by oxidation, starting with the preparation of the radical cation of EDOT monomer, which attacks a neutral EDOT, followed by deprotonation. It has optical transparency in its conducting state, high stability, and moderate band gap and low redox potential [120,121]. PEDOT nanotubes with interfacial conducting properties were successfully utilized for neural recording [122]. Richardson-Burns et al. [123] demonstrated the electrochemical polymerization of PEDOT around living neuronal cells with no toxic effects. Furthermore, PEDOT combined with poly(styrene-sulfonate) (PSS) (PEDOT:PSS) has proven to be an excellent system with good conductivity, good stability, high optical transparency, and low toxicity. Thus, it is widely used in electronic circuits, electrostatic packaging, OLEDs, sensing, and photovoltaic devices [124,125,126]. Yang et al. [127] prepared silver nanowire (AgNW)-PEDOT:PSS composite transparent flexible electrodes (FTEs) through a Mayer rod coating method. The AgNW-PEDOT:PSS composite FTEs exhibited high optoelectrical properties, with a sheet resistance of 12 Omega sq^−1^ and a transmittance of 96% at 550 nm. Unfortunately, the biodegradability of PEDOT is also low.

In general, melanin is the only conductive polymer that exhibits both biodegradability and biocompatibility, but its availability and mechanical properties are not sufficient, which limits its broad application. Synthesized polymers with electric properties, including PANI, PPy, and PEDOT, are biocompatible but not biodegradable. Thus, the application of bare (semi)conductive biopolymers in electronic devices is greatly limited. To obtain (semi)conductive biomaterials with excellent biodegradability, incorporating conductive nanofillers into biodegradable biopolymers is an efficient solution. This field of research will be carefully reviewed in the following section.

## 3. Applications of Nanocomposites for Electronics

### 3.1. Substrates

**Figure 2:**
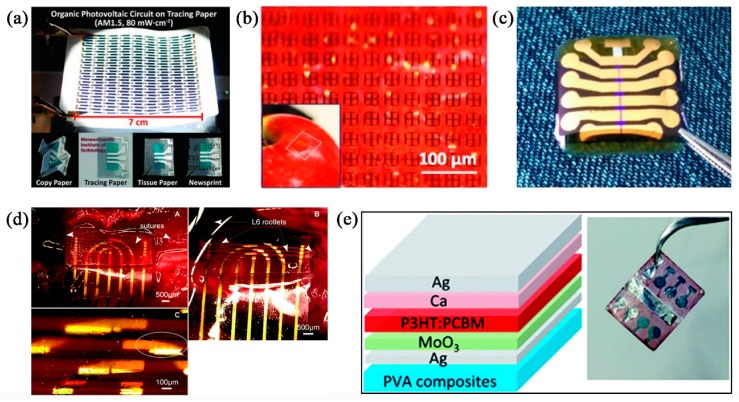
(**a**) Organic photovoltaic circuits fabricated on various paper substrates. Reproduced with permission from [60], Wiley-VCH, 2011. (**b**) split ring resonators fabricated on the silk substrate, wrapped on an apple. Reproduced with permission from [69], Wiley-VCH, 2012. (**c**) biodegradable transistors fabricated on shellac substrate. Reproduced with permission from [80], Wiley-VCH, 2012. (**d**) pressure sensor with polydimethylsiloxane (PDMS) substrate, mounted on rat spinal cord. Reproduced with permission from [41], RSC, 2012. (**e**) schematic device structure and optical image of the transient organic solar cells with poly(vinyl alcohol) (PVA) substrates. Reproduced with permission from [82], RSC, 2017.

Electronic devices usually consist of a solid substrate and several functional components, such as semiconducting layers, dielectric layers, electrodes, and capsulations. All of these components can be fabricated with biopolymers, replacing the traditional polymers, which are not environmentally friendly. The substrate’s role is to support other layers. As such, it is thicker and larger, and generates more E-waste. It also commonly electrically isolates the electronics to prevent undesirable crosstalk, and thus the substrate material is usually insulated. Figure 2 shows some electronic devices whose substrates were fabricated with different insulated biodegradable or biocompatible polymers.

Flexibility is of paramount importance for the substrates of stretchable electronics. Generally, adding nanofillers into substrate materials would seriously decrease the flexibility of the substrate, thus, substrate material is usually pure polymer without nanofillers. The most common biodegradable nanomaterial applied in substrates is nanocellulose (NC). Depending on the preparation methods, NCs can be classified as cellulose nanocrystals (CNCs) or cellulose nanofibrils (CNFs) [128,129,130]. NCs possess a large variety of superior characteristics, such as biodegradability, environmental sustainability, inherent renewability, simplified disposal, distinctive morphology, outstanding chemical-modification capabilities, and extraordinary mechanical strength [128,131,132,133,134,135]. They have attracted great attention in recent years [131,136,137,138,139]. Because of their diverse properties, morphologies, and forms, NCs have great potential in a variety of applications, including biomaterial engineering, batteries and solar cells, textiles and clothing, food, packaging industries, and electronic devices [140,141,142,143,144].

**Figure 3:**
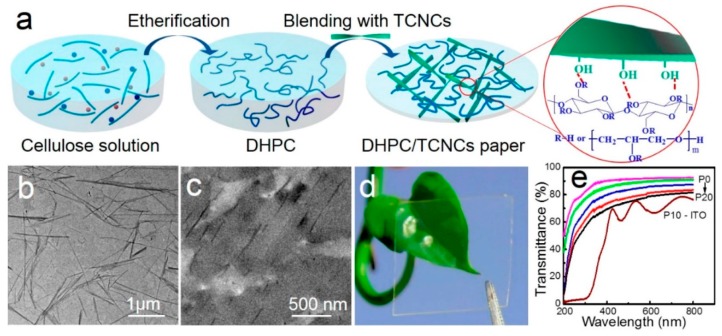
(**a**) The fabrication process of cellulose-based nanocomposite papers; (**b**) transmission electron microscope (TEM) image of tunicate cellulose nanocrystals (TCNCs); (**c**) TEM image of P10; (**d**) photograph of P10; (**e**) optical transmittance of neat *O*-(2,3-Dihydroxypropyl) cellulose (DHPC) and nanocomposite papers under UV-vis light. Reproduced with permission from [146], ACS, 2018.

In the field of electronics, many devices with NCs as substrates have been reported. Park et al. [145] displayed a flexible, transparent, and nontoxic phototransistor for detecting visible light, which was fabricated on biodegradable CNF substrates. They carried out mechanical bending tests with radii ranging from 100 to 5 mm and cyclic bending tests of up to 2000 cycles at a fixed radius of 5 mm. The bending test proved excellent operational stability. Combined with the phototransistors’ flexibility, transparency, and biodegradability, this report indicates the significant potential of NCs as low-cost and environmentally friendly sensors. Cheng et al. [146] synthesized *O*-(2,3-Dihydroxypropyl) cellulose (DHPC) by the homogeneous etherification of cellulose in 7 wt.% NaOH/12 wt.% urea aqueous solution, and then introduced stiff tunicate cellulose nanocrystals (TCNCs) into the DHPC, in order to construct tough nanocomposite papers. Owing to the excellent interfacial compatibility between TCNCs and DHPC, the nanocomposite papers had smooth surfaces, high transparency, and excellent mechanical properties, enabling them to be used as the substrates of biodegradable and wearable electronics. A fabrication schematic of the cellulose-based nanocomposite papers and their properties is shown in Figure 3.

**Figure 4:**
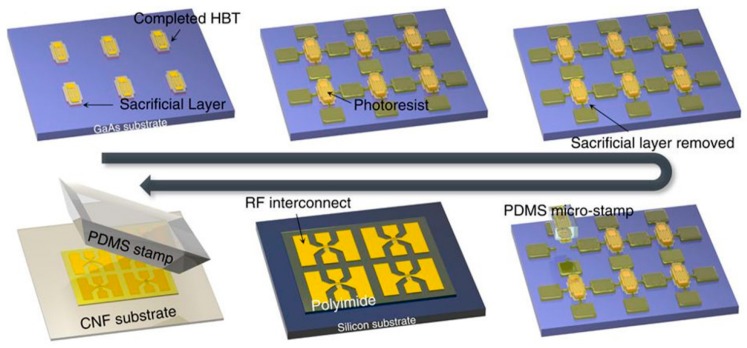
The fabrication process of GaAs devices built on cellulose nanofibril (CNF) paper. Reproduced with permission from [147], NPG, 2015.

Jung et al. [147] utilized biodegradable and flexible CNF papers as substrates and constructed many electronic devices, including flexible microwave and digital electronics, gallium arsenide microwave devices, and consumer wireless workhorses. Figure 4 shows the fabrication process of GaAs devices built on CNF papers.

### 3.2. Conductors and Semiconductors

Many biodegradable or biocompatible polymers, including melanin, PANI, PPy, and PPDOT can be used as conductors or semiconductors in electronic devices. Nevertheless, the conductivities of these polymers are usually not sufficiently efficient. To address this problem, some functional nanofillers have been incorporated into conductive polymers to improve their conductivities. Nanofillers have even been added into insulated polymers to endow them with conductivities [148,149]. Among these nanofillers, graphene and carbon nanotubes (CNTs) are most widely used [150,151,152].

Graphene is a single layer of hybridized carbon atoms arranged in a two-dimensional lattice, which can be manufactured by peeling graphite nanosheets. It has outstanding thermal, optical, mechanical, and electrical properties, attributed to its special structure [153,154,155,156]. The carrier mobility of graphene at room temperature is about 15,000 cm^2^ V^−1^ s^−1^, 10 times higher than that of silicon. The excellent conductivity makes it an efficient nanofiller to improve the electrical conductivity of polymers.

**Figure 5:**
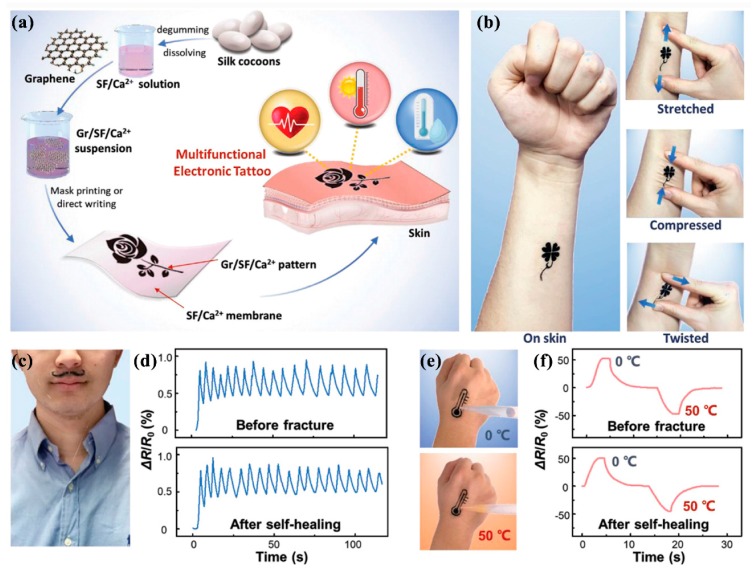
(**a**) The fabrication illustration of a Gr/silk fibroin (SF)/Ca^2+^ E-tattoo; (**b**) E-tattoo mounted on the forearm and its variations in stretched, compressed, and twisted states; (**c**) E-tattoo mounted on the upper lip for monitoring respiration; (**d**) comparison of the relative resistance between unbroken and healed humidity sensors; (**e**) E-tattoo mounted on the hand for monitoring temperature; (**f**) ccomparison of relative resistance between unbroken and healed temperature sensors. Reproduced with permission from [157], Wiley-VCH, 2019.

Wang et al. [157] reported a healable and multifunctional E-tattoo based on a graphene/silk fibroin (SF)/Ca^2+^ combination. The flexible E-tattoos are fabricated by printing or writing with a graphene/SF/Ca^2+^ suspension. The graphene sheets are uniformly distributed in the matrix, forming an electrically conductive path, which can sensitively respond to the changes of the surrounding environment, including strain, temperature, and humidity. This property enables the E-tattoo to be used as a sensor, monitoring these variables with high sensitivity, fast response, and excellent stability. In addition, the E-tattoo exhibits excellent healable properties. After being damaged by water, the E-tattoo can remarkably and completely heal in only 0.3 s, as a result of the effective reformation of hydrogen and coordination bonds at the fractured interface. The fabrication process and applications of E-tattoos as humidity and temperature sensors are shown in Figure 5.

Ling et al. [158] also utilized graphene to develop novel conductive nanocomposites and to fabricate sensors. They used SF as a matrix and prepared graphene/SF nanocomposites through a uniformly dispersed and highly stable graphene/SF suspension system. The prepared graphene/SF nanocomposites maintain not only the electronic advantages of graphene but also the mechanical properties of SF. Their electrical resistances are sensitive to deformation, body movement, humidity, and changes in the chemical environment, showing a promising future for effective applications as wearable sensors, intelligent skins, and human–machine interfaces.

Similarly, Scaffaro et al. [159] prepared a piezoresistive sensor by exploiting amphiphilic graphene oxide (GO) to endow the polylactide (PLA)-poly (ethylene-glycol) (PEG) blends with electrical properties sensitive to changes in pressure and strain. The responsivity of the biodegradable pressure sensor is 35 μA MPa^−1^ from 0.6 to 8.5 MPa, and 19 μA MPa^−1^ from 8.5 to 25 MPa, while in lower pressure ranges (around 0.16–0.45 MPa) the responsivity reaches 220 μA MPa^−1^. Additionally, the presence of GO acts as a compatibilizer, providing stiffness and strength without any negative impact on toughness. It provides the stability of mechanical properties for up to 40 days.

CNTs, as one-dimensional nanomaterials, have abnormal mechanical, electrical, and chemical properties. Recently, in an in-depth study, CNTs revealed broad prospective applications. CNTs are formed by crimping graphene sheets, and can be classified into single-walled CNTs (SWCNTs) and multi-walled CNTs (MWCNTs), according to the number of layers of the graphene sheets [160]. The P-electrons of the carbon atoms on CNTs form a wide system of delocalized π bonds, endowing CNTs with special electrical properties due to the significant conjugation effect. According to theoretical prediction, the conductivity of CNTs depends on their diameter and the helical angle of the wall. When the diameter of CNTs is greater than 6 nm, their conductivity is relatively lower; when less than 6 nm, CNTs can be regarded as one-dimensional quantum wires with good conductivity. Their excellent properties make CNTs desirable for high-strength, conductive nanocomposites based on sustainable resources and polymer materials [161,162]. Many efforts have been made to utilize CNTs by preparing conductive nanocomposites and then using them in biodegradable electronic devices [163,164,165,166,167,168].

**Figure 6:**
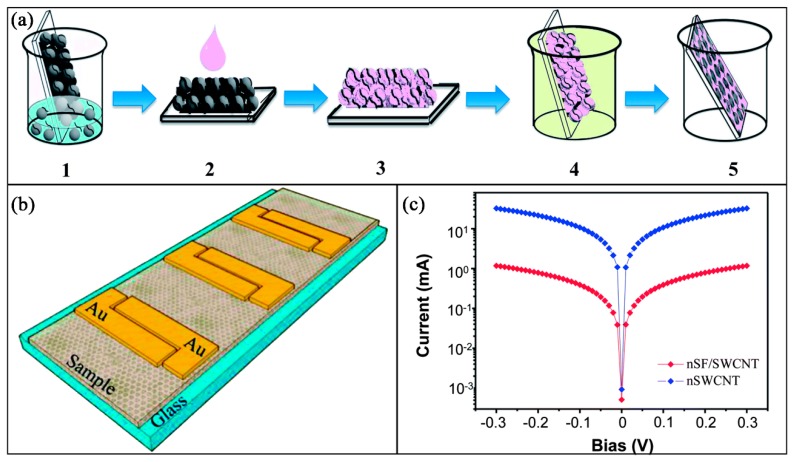
(**a**) Schematic illustration of the fabrication process of the porous SF-SWCNT films; (**b**) device, including two gold pads which contact the film; (**c**) comparison of the I-V characteristics at 0.3 V for the SF-SWCNT and SWCNT films. Reproduced with permission from [169], RSC, 2014.

Dionigi et al. [169] prepared a conductive nanocomposite with SF and SWCNTs using a novel wet templating method, which combines the excellent mechanical properties and biocompatibility of SF with the electric conductivity and stiffness of SWCNTs. The prepared SF-SWCNT nanocomposites exhibit a periodic structure in which SWCNTs are regularly and uniformly distributed in the SF matrix. The film based on the SF-SWCNT nanocomposites possesses a conductivity only one order of magnitude lower than the bare SWCNTs. Remarkably, the SF-SWCNT nanocomposite enables the growth of primary rat Dorsal Root Ganglion neurons. Figure 6 displays the fabrication process of the nSF-SWCNT film and its electrical properties.

Sivanjineyulu et al. [170] prepared poly(butylene succinate) (PBS)/PLA blend-based nanocomposites with CNTs as reinforcing nanofillers. The electrical resistivity values of PBS, PLA, or their blends are all higher than 1013 Ω square^−1^, illustrating that they are electrically insulated. When CNT is added into the PBS/PLA blend, the electrical resistivity is greatly decreased. Even with only a 3 phr CNT loading, the electrical resistivity of the blend decreased by up to 11 orders of magnitude as a result of the formation of a semi-conductive network structure in the nanocomposite system.

Valentini et al. [168] reported a photo-responsive device with a semiconducting polymer film built on semitransparent and conductive biodegradable poly(3-hydroxybutyrate) (PHB)/CNT substrates. The biodegradable PHB/CNT nanocomposite can be prepared using SWCNTs or MWCNTs through a simple solvent-casting approach. The electrical resistance value measured on the PHB-SWCNT sample is around 3 × 10^7^ Ohm, while that on the PHB-MWCNT sample is around 2 × 10^8^ Ohm.

**Figure 7:**
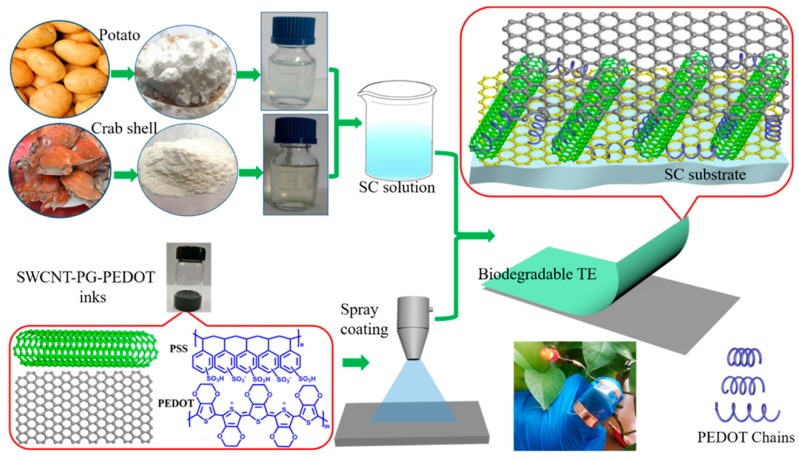
Fabrication process of the biodegradable and flexible 3D interconnected single-walled carbon nanotubes-pristine graphene-poly(3,4-ethylenedioxythiophene) (SWCNT-PG-PEDOT) based transparent electrode. Reproduced with permission from [55], ACS, 2018.

In addition to adding graphene or CNTs as nanofillers into polymer matrices independently, some researchers also added both graphene and CNTs simultaneously into a polymer’s matrix. For example, Miao et al. [55] reported a biodegradable and flexible transparent electrode, in which the prepared conductive nanocomposites had a 3D interconnected SWCNT-pristine graphene (PG)-PEDOT network architecture and was structured using Nacre-inspired interface designs. The one-dimensional SWCNT and the two-dimensional PG sheets were tightly cross-linked at the junction interface by PEDOT chains. The fabrication process of the transparent electrode is shown in Figure 7. The formation of the SWCNT-PG-PEDOT continuously conductive network results in a low electrical resistance, as well as excellent flexibility. Even after hundreds of bending cycles, the electrical resistance of the electrode only increases by less than 3%. Moreover, the fabricated electrode exhibits an outstanding optoelectronic property: typically, a sheet resistance of 46 Ω square^−1^ with a transmittance of 83.5% at a typical wavelength of 550 nm. More importantly, the conductive nanocomposites are incorporated with an edible starch-chitosan substrate, which leads to perfect biodegradability: it could be rapidly degraded in a lysozyme solution at room temperature, with no toxic residues produced.

Chen et al. [171] prepared Polycaprolactone (PCL)/MWCNT nanocomposites by blending GO sheets and MWCNTs into PCL, where GO acts as an adjuvant for regulating the dispersion state of MWCNTs and thus balances the electrical and mechanical properties of the nanocomposites. Strong π-π interactions between MWCNTs and GO nanosheets make it easy for MWCNTs to be adsorbed onto the surfaces of GO nanosheets, thereby forming GO/MWCNT hybrids, which hinder the aggregation of MWCNTs in PCL. Based on this mechanism, the dispersion of GO/MWCNT hybrids in PCL is greatly affected by the GO/MWCNT ratio. The dispersion states of MWCNTs in PCL were divided into PCL/MWCNT, PCL/GO/MWCNT (1/4), and PCL/GO/MWCNT (2/1) in their research, representing severe, low, and almost no aggregation of MWCNTs, respectively. Among the three dispersion states, the PCL/GO/MWCNT nanocomposites with a GO/MWCNT ratio of 2/1 showed the best MWCNT dispersion in PCL matrix, and thus the highest tensile strength and elongation at break. However, the PCL/GO/MWCNT (1/4) nanocomposites achieved the best electrical conductivity. This is attributed to the relatively low MWCNT aggregation.

**Figure 8:**
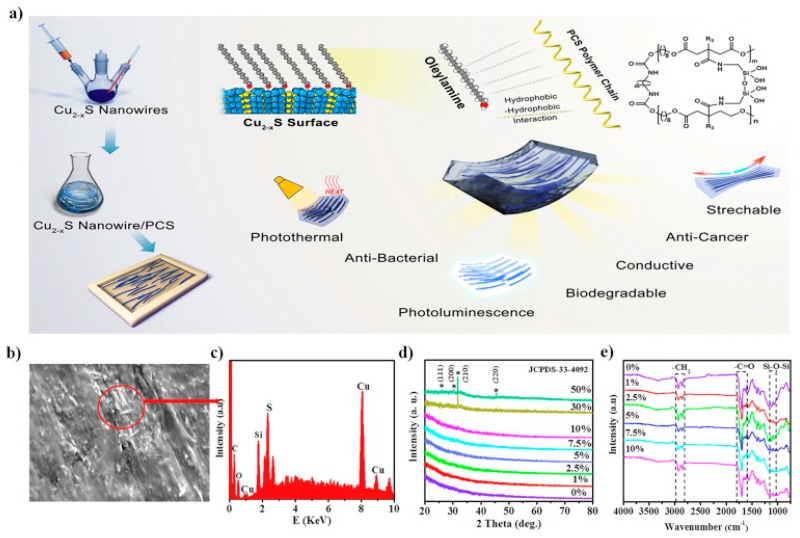
A processing illustration and physicochemical structure characterizations of the poly(citrates-siloxane)-copper sulfide nanowires (PCS-CSNW) nanocomposites: (**a**) processing illustration and potential applications for biomedicine; (**b**) scanning electron microscope (SEM) images; (**c**) energy dispersion spectrum (EDS) spectrum; (**d**) X-ray diffraction (XRD) patterns; (**e**) Fourier transform infrared (FTIR) spectra between 4000 and 650 cm^−1^. Reproduced with permission from [172], Elsevier, 2019.

**Other nanofillers.** Aside from carbon nanotubes and graphene, metal nanowires can also improve the conductivity of the nanocomposites. Li et al. [172] reported biodegradable poly(citrates-siloxane) (PCS) elastomers reinforced by ultralong copper sulfide nanowires (CSNWs). The CSNWs were uniformly distributed throughout the PCS matrix because of the hydrophobic-hydrophobic interaction between them. The content of the CSNWs directly influences the electric conductivity of the nanocomposites, which could reach a high value of 5 × 10^−4^ S cm^−1^ when the addition content of CSNWs is 30%. PCS-CSNW also exhibits a high degree of biocompatibility, which decreases the inflammatory reaction of cells. Additionally, it possesses a unique photo-luminescent property and strong near-infrared (NIR) photo-thermal capacity, which allows in vivo thermal imaging and biodegradation tracking with high resolution. PCS-CSNW could assist in the effective killing of cancer cells via a selective NIR-induced photo-thermal therapy [173]. Therefore, PCS-CSNW is a promising material in the area of next-generation implanted electronics, tissue engineering or regenerative medicine for biomedical applications. A processing illustration and physicochemical structure characterizations of the PCS-CSNW nanocomposites are shown in Figure 8.

**Figure 9:**
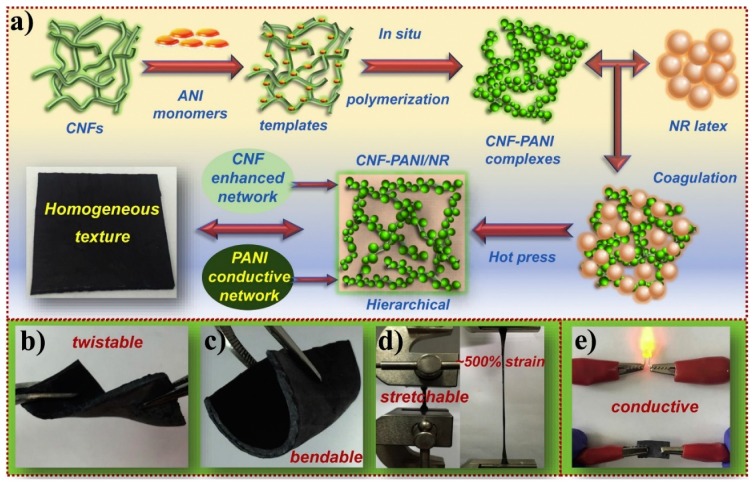
The processing illustration of the conductive cellulose nanofibril-polyaniline/natural rubber (CNF-PANI/NR) elastomers and their properties: (**a**) processing illustration of the conductive CNF-PANI/NR hybrid elastomers; (**b**) flexibility of the CNF-PANI/NR elastomers; (**c**) bendability of the CNF-PANI/NR elastomers; (**d**) stretchability of the CNF-PANI/NR elastomers; (**e**) conductivity of the CNF-PANI/NR elastomers. Reproduced with permission from [112], Elsevier, 2019.

Not all nanofillers in conductive nanocomposites are designed to improve electrical properties. They may also be used only to strengthen materials, and electrical conductivity is instead achieved by conductive polymers. Han et al. [112] reported conductive hybrid elastomers fabricated with a natural rubber (NR) matrix and nanostructured CNF-PANI complexes, in which PANI provides the conductivity, while CNFs strengthen the material. The CNF-PANI complexes were prepared via oxidative polymerization of aniline monomers on CNF surface, and then evenly dispersed into NR latex to fabricate CNF-PANI/NR elastomers using latex co-coagulation. The presence of CNFs in the nanocomposites constructs a reinforcing network and simultaneously supports the 3D conductive network in NR matrix. The fabricated bio-based elastomers with homogeneous structures showed inherent flexibility, improved mechanical properties, decent stretchability, low density, and desired electric conductivity (up to 8.95 × 10^−1^ S m^−1^). Then, the elastomer was used to fabricate a strain sensor with high sensitivity and repeatability, which could monitor the motion of the human body in real time. The elastomer-based electrode with 20 phr of PANI presented superior electrochemical properties. Its specific capacitance could reach a maximum of 110 F g^−1^ with a relatively low capacitance degradation of 22% after 1200 cycles at a current density of 0.3 A g^−1^. The processing illustration of the conductive CNF-PANI/NR elastomers and their properties are shown in Figure 9.

Aside from the nanocomposites previously discussed, there are many other biodegradable and biocompatible nanocomposites used to enhance electronic conductors, which will not be carefully discussed here [174,175,176,177,178,179,180].

### 3.3. Dielectrics

Dielectric materials are usually electrically insulated and can be polarized when an electric field is applied. Under the action of an electric field, the electric charges in dielectric materials will slightly deviate from their equilibrium positions, rather than flowing and forming current as in conductive materials. The slight movement of positive and negative charges produces an internal electric field opposite to the direction of the applied eternal electric field, thereby reducing the total electric field in the dielectric material. For example, the dielectric layer in OFETs produces induced electric charges in the semiconducting channel when the gate voltage is applied. The dielectric constant and breakdown voltage of the dielectric layer are two crucial parameters that must be carefully considered for low-voltage operation and long-time stability [1].

Many naturally-based and synthesized substrate materials can be used to make dielectrics, such as cellulose, silk, shellac, gelatin, PVA, PDMS, PGS, Poly(lactic-co-glycolic acid) (PLGA), PCL, and PLA. Regarding dielectric polymers, adding a small amount of nanofillers into them to prepare nanocomposites would further enhance their dielectric performance. The chemical structure, surface morphology, and preparation method, as well as the additives, all have an effect on the dielectric properties of the nanocomposites [39,181,182,183]. Conductive and high dielectric constant nanofillers, such as CNTs, GO, Al_2_O_3_, and SiO_2_, all have the potential to improve dielectric performance. Kashi et al. [184] investigated the effect of graphene nanosheets on the dielectric performance of biodegradable nanocomposites and found that the presence of graphene nanosheets could heighten the dielectric constant of polymers to a large extent.

Deshmukh et al. [185] prepared bio-based nanocomposites by blending cellulose acetate (CA) with Al_2_O_3_ nanoparticles (Al_2_O_3_ NPs) and investigated the microstructure, morphology, thermal, and dielectric properties of the CA/Al_2_O_3_ nanocomposites. In the solution blending process, the Al_2_O_3_ NPs were uniformly dispersed in the CA matrix and showed good intermolecular interaction. The incorporation of Al_2_O_3_ NPs significantly enhanced the dielectric properties of CA. For instance, in the condition of 50 Hz and 30 °C, when loaded with 25 wt.% Al_2_O_3_, the dielectric constant increased from 8.63 to 27.57 and the dielectric loss increased from 0.26 to 0.64. However, the values of tan δ for all the samples were all very low (below 1).

**Figure 10:**
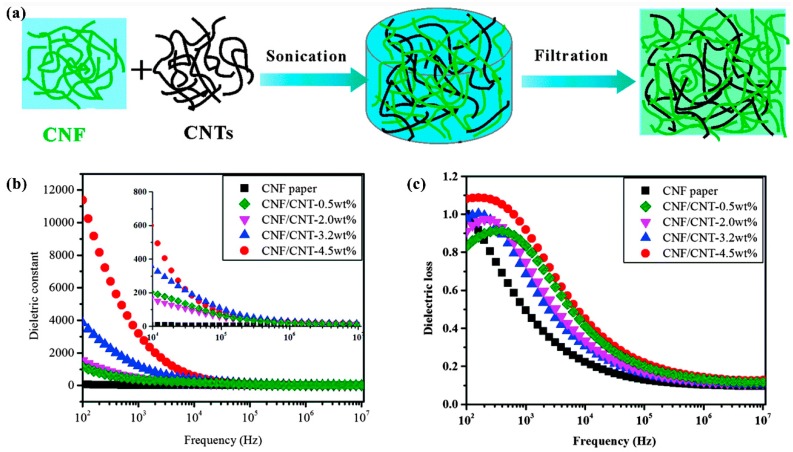
(**a**) Preparation process of the CNF/CNT papers; (**b**,**c**) frequency dependence of dielectric constant and loss of the CNF/CNT papers with different CNT loadings. Reproduced with permission from [186], RSC, 2016.

Zeng et al. [186] reported flexible dielectric papers based on biodegradable CNFs and CNTs for dielectric energy storage. They successfully prepared highly ordered, homogeneous CNF/CNT papers through a simple vacuum-assisted self-assembly technique. When the CNT loading was 4.5 wt.%, the dielectric constant of the CNF/CNT paper was 3198 at a frequency of 1.0 kHz, which is far higher than 15 for the neat CNF paper. The significant enhancement resulted from the formation of microcapacitor networks in the papers by neighboring conductive CNTs and insulating CNFs. The excellent dielectric constant also improved the dielectric energy storage capability (0.81 ± 0.1 J cm^−3^). In addition, the CNF/CNT papers showed a high degree of flexibility and enhanced mechanical strength. The preparation process and dielectric properties of the CNF/CNT papers are shown in Figure 10.

Choudhary [187] prepared polymer nanocomposite films with a biodegradable polymer blend matrix of PVA and poly(vinyl pyrrolidone) (PVP) and dispersed amorphous silica (SiO_2_) nanoparticles using the aqueous solution-cast method. It was found that the presence of the dispersed SiO_2_ nanoparticles in the PVA–PVP blend matrix decreased the size of PVA crystallites, and forced the surface morphology of the nanocomposite films to turn from smooth to relatively rough. The dielectric constant of the nanocomposite films decreased as the SiO_2_ content increased to 3 wt.%. However, when the SiO_2_ content was 5 wt.%, the dielectric constant was close to that of the pure polymer blend matrix. Additionally, temperature had an effect on the dielectric constant. The dielectric constant of the nanocomposite film increased non-linearly with the increase of temperature.

Deshmukh et al. [188] also prepared SiO_2_ nanoparticle-reinforced PVA and PVP blend nanocomposite films. SiO_2_ nanoparticles were homogeneously dispersed in the PVA/PVP blend matrix in a solution-blending process. The dielectric constant and dielectric loss of the PVA/PVP/SiO_2_ nanocomposite films were tested under a frequency range of 10^−2^ Hz to 20 MHz and temperature range of 40–150 °C. In the testing conditions, the dielectric constants of the prepared nanocomposites were higher than those of PVA/PVP blends. With 25 wt.% SiO_2_ content, the dielectric constant reached a maximum of 125 (10^−2^ Hz, 150 °C) and the dielectric loss was 1.1 (10^−2^ Hz, 70 °C). Deshmukh et al. obtained better dielectric properties compared to Choudhary [187], with the same PVA/PVP/SiO_2_ system. The results reported by Deshmukh et al. show that SiO_2_ could significantly improve the dielectric properties of polymers, and the solution-casting method they utilized has great potential for flexible organic electronics.

Deshmukh et al. [183] fabricated flexible dielectric nanocomposites, which are composed of water soluble PPy (WPPy), PVA, and GO, and then characterized them at different GO contents (0.5–3 wt.%). Because of the presence of GO and its uniform dispersion in the polymer matrix, the nanocomposites show a significant improvement in the dielectric constant with low dielectric loss. With a GO loading of 3 wt.%, frequency of 50 Hz, and temperature of 150 °C, the dielectric constant increased from 27.93 for WPPy/PVA blend to 155.18 for nanocomposites, and the dielectric loss increased from 2.01 for WPPy/PVA blend to 4.71 for nanocomposites.

As can be seen from the above references in this section, adding nanofillers, such as CNTs, GO, Al_2_O_3_, and SiO_2_, into polymers can enhance the dielectric properties of those polymers. With biocompatible and biodegradable polymer matricies, the newly developed nanocomposites would enable the feasible fabrication of dielectrics with high-performance capabilities, flexibility, and environmental friendliness.

## 4. Electronics Packaging

Unlike substrates, (semi)conductors, or dielectrics, materials for electronics packaging require different functions for various operational environments, such as cyclical mechanical bending, aqueous solutions, elevated temperatures, electromagnetic shielding, electrostatic prevention. Most polymeric substrate materials, such as PLGA, PCL, parylene-c, and poly(vinyl acetate) PVAc, can be used to form strain-resistant packaging layers to prevent the rapid degradation of devices [52,189,190,191,192].

One particular concern is to ensure that the packaging materials are able to repel gas and water vapor over a period of several months, because many conjugated organic compounds used as (semi)conductors are easy to oxidize and lose their function in ambient and aqueous environments. Therefore, the gas and water vapor barrier property is the most important consideration in the packaging of electronics. Biodegradable polymers are likely be used to meet this demand due to their high crystallinity, high hydrophobicity, and facile processing [193]. An adequate packaging polymer is poly(L-lactide) (PLLA), which satisfies the aforementioned demand and can be easily prepared by melt casting or using ordinary organic solvents. Adding nanofillers into polymers is an efficient method to enhance gas and vapor barrier properties, and many nanofillers, including organophilic layered double hydroxides (OLDH) nanosheets [194,195,196], montmorillonite [197], and GO [198], have been used for this purpose.

**Figure 11:**
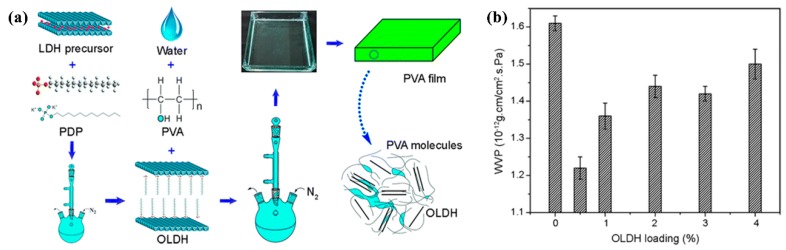
(**a**) Processing of the PVA/organophilic layered double hydroxides (OLDH) films; (**b**) water vapor permeability variation as a function of OLDH loading. Reproduced with permission from [194], Springer, 2017.

Xie et al. [194] incorporated OLDH nanosheets into a biodegradable PVA matrix via a solution casting method and prepared PVA/OLDH films. The OLDH nanosheets, which were intercalated with aliphatic long-chain molecules as reinforcing agents, were homogeneously dispersed in PVA matrix and formed strong interfacial interactions with the PVA chains, resulting in significant enhancements of optical property, mechanical performance, thermal stability, and water vapor barrier property. Even when only 0.5 wt.% OLDH was loaded in PVA, water vapor permeability could decrease by 24.22%. The significant improvement of the water vapor barrier property results from the homogeneous dispersion of OLDH nanosheets, which causes the paths for water vapor diffusion to be tortuous and thus decreases the water vapor permeability. The experiments demonstrated that the PVA/OLDH nanocomposite films have a wide variety of potential applications in the field of electronics packaging. A schematic illustration for the preparation of PVA/OLDH films is shown in Figure 11.

**Figure 12:**
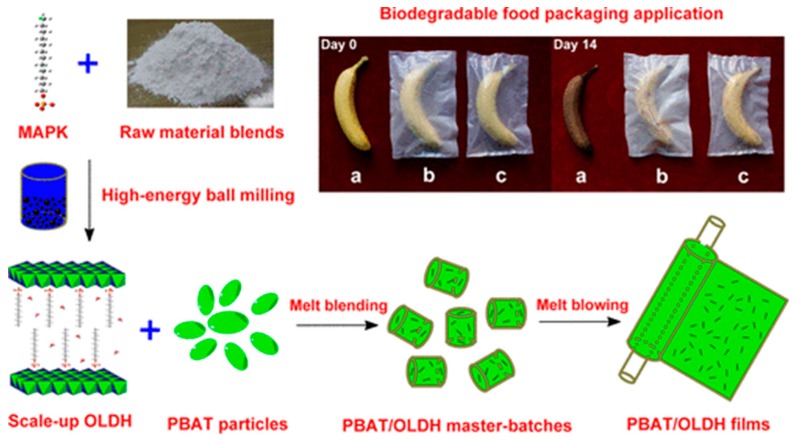
Fabrication process for the poly(butylene adipate-co-terephthalate) (PBAT)/OLDH nanocomposite films. Reproduced with permission from [195], ACS, 2018.

Xie et al. [195] synthesized a series of biodegradable nanocomposite films based on poly(butylene adipate-co-terephthalate) (PBAT), and reinforced them with OLDH nanosheets. The OLDH nanosheets were pre-synthesized by solvent-free high-energy ball milling and dispersed uniformly in the PBAT matrix. Compared with pure PBAT films, PBAT/OLDH films with 1 wt.% OLDH loading exhibited improved thermal, optical, mechanical, and water vapor barrier properties, including a 37% reduction in haze and a 41.9% increase in nominal tensile strain at break. The feasibility of scale-up production, outstanding processability, manufacturing scalability, mechanical property, optical transparency, and water vapor barrier properties indicate a promising future application of the PBAT/OLDH nanocomposite films as biodegradable packaging films. Figure 12 shows the schematic illustration of the fabrication process for the PBAT/OLDH nanocomposite films.

Aside from the OLDH nanosheets, montmorillonite is another commonly used nanofiller for the improvement of barrier performance. Wang and Jing [197] prepared biodegradable montmorillonite/chitosan nanocomposites and coated them onto the traditional package paper so as to expand the potential application of the paper. They found that montmorillonite/chitosan nanocomposite showed superior water vapor barrier properties, especially with a high montmorillonite and dispersant content, dispersion rate, and coating weight. In addition, the montmorillonite/chitosan nanocomposite coated with a lower content of montmorillonite or with a higher dispersion speed and dispersant content had better smoothness and elongation. However, the addition of OLDH nanosheets had a bad impact on the formation process.

GOs can also be used as nanofillers for enhancing the gas and water vapor barrier performance of polymer systems. Ren et al. [198] introduced an extremely low amount of GO nanosheets into biodegradable poly(butylene adipate-co-terephthalate) (PBAT) and the barrier performance was significantly improved. The permeability coefficients of oxygen and water vapor decreased, exceeding 70% and 36% with a GO nanosheet loading of 0.35 vol.%. The enhanced barrier performance was attributed to the excellent impermeability and homogeneous dispersion of GO nanosheets, as well as the strong interfacial adhesion between the GO nanosheets and PBAT matrix.

**Figure 13:**
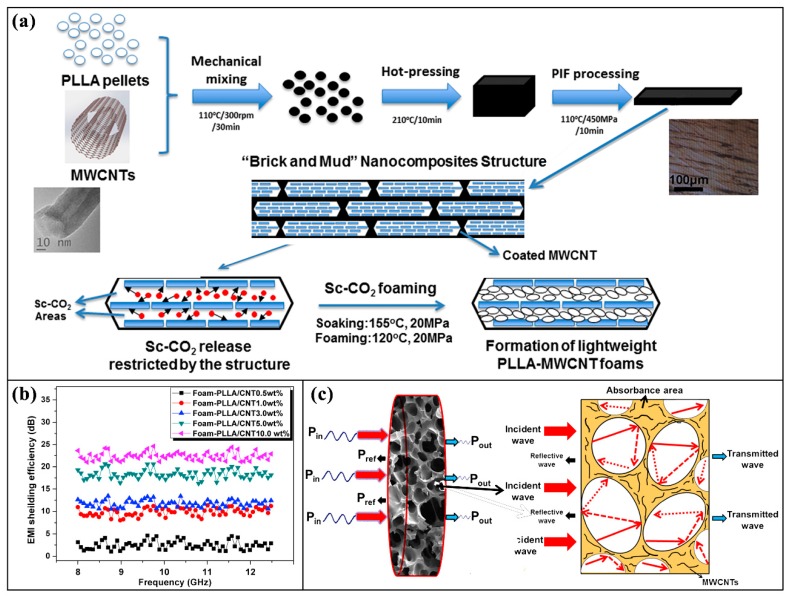
(**a**) Fabrication process of the lightweight poly(L-lactide)/single-walled carbon nanotubes (PLLA/MWCNT) nanocomposite foams using a combinatorial technology of pressure induced flow processing and Sc-CO_2_ foaming; (**b**) electromagnetic interference (EMI), shielding efficiency (SE) of PLLA/MWCNT nanocomposite foams in the frequency ranges of 8.00–12.48 GHz; (**c**) schematic illustration of electromagnetic microwave dissipation in the PLLA/MWCNT nanocomposite foams. Reproduced with permission from [201], Elsevier, 2016.

In addition to having a gas and water barrier, some special electronic devices need be packaged with materials having electromagnetic shielding and antistatic functions. In the electromagnetic shielding field of electronics packaging, nanocomposites have great advantages compared with pure polymers because of the addition of conductive nanofillers [199,200,201,202]. For example, Kuang et al. [201] developed lightweight high-strength PLLA/MWCNT nanocomposites foams with an efficient, environmentally friendly, and inexpensive method, using a pressure-induced flow technique and solid-state supercritical CO_2_ foaming. The nanocomposite foams have a density as low as 0.3 g cm^−3^, possess an electric conductivity of 3.4 S m^−1^, and an electromagnetic interference (EMI) shielding efficiency (SE) of around 23 dB in the range of 8.00–12.48 GHz. The corresponding average specific EMI SE reaches 77 dB g^−1^ cm^3^, far exceeding those of metals and many carbon-based composites with similar densities and thickness. Absorption was proven to be the major mechanism of EMI shielding for the PLLA/MWCNT nanocomposite foams, which is shown in Figure 13. In addition, the nanocomposite foams also show superior compressive stress. The prepared biodegradable PLLA/MWCNT nanocomposites foams are suitable for EMI shielding in electronics packaging.

**Figure 14:**
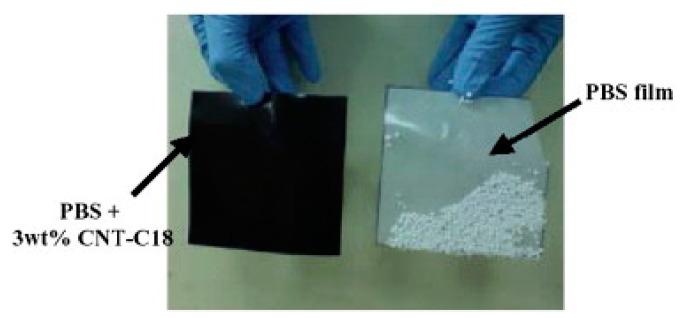
Anti-static test by Shih et al. Reproduced with permission from [203], Elsevier, 2008.

In antistatic aspects of packaging, nanocomposites also have great advantages compared with pure polymers. Shih et al. [203] prepared PBS/MWCNT nanocomposites through a melt–blending method. The MWCNTs were firstly modified with *N,N’*-dicyclohexylcarbodiimide (DCC) dehydrating agents, and then uniformly dispersed in organic solvents. The PBS/MWCNT nanocomposites were subsequently prepared via the facile melt–blending process. The prepared PBS/MWCNT nanocomposites exhibit a surface resistivity of 7.30 × 10^6^ Ω, 10^9^ folds lower in value compared with the neat PBS sample. At an MWCNTs loading of 3 wt.%, the PBS/MWCNT nanocomposites showed an excellent anti-static capacity, indicating a promising potential in electronic packaging materials for anti-static function. Figure 14 shows the results of anti-static test.

## 5. Summary and Outlook

Biodegradable nanocomposites have been widely investigated and used for fabricating components of green electronics, and provide an efficient solution for E-waste management and environment protection. Furthermore, green electronics also exhibit promising potential for biomedical applications of transient electronic devices. Although functional nanomaterials have significantly enhanced the overall performances of biodegradable polymers, some particular characteristics, including electric conductivity and flexibility, as well as biodegradability and biocompatibility, still need be further improved.

Flexibility mainly depends on the properties of the polymer matrix. Synthetic polymers display superiority in flexibility, as well as better conformal contact between the implantable electronics and dynamic tissue surface, but they generally suffer from worse biodegradability or biocompatibility. To achieve an excellent performance for all the requirements still remains an arduous challenge. Developing novel polymers derived from natural materials with enhanced mechanical properties or blending the natural-based polymers and synthetic polymers together may be feasible methods for future improvement.

Electric conductivity is affected by both the polymer matrix and nanofillers. Synthetic conjugated polymers are usually not biodegradable, and thus a conjugation-breaking degradation strategy through mechanisms such as oxidation, ultraviolet (UV) exposure, or enzymes, without affecting conductivities is crucial. However, the trade-off between material degradability and device stability is difficult to balance. Natural-based conductive polymers can be easily biodegradable, but they suffer unsatisfied conductivity and bad mechanical properties, which seriously limits their applications. Adding functional nanofillers, such as graphene and CNTs, into a polymer matrix can significantly enhance the electric conductivity. The electric conductivity is deeply affected by the dispersion state of nanofillers in the polymer matrix as well as the interfacial morphology, which has been studied by many researchers and will still be an important research direction.

## Figures and Tables

**Table 1:** Summary of biopolymers mentioned in this review.

Category	Polymer Material	Electrical Property	Biodegradable/Biocompatible	Applications
Natural Polymers	Cellulose	Insulator	Both	Substrate [24,25]; Dielectric [26]
	Silk	Insulator	Both	Substrate [27,28]; Dielectric [29]
	Shellac	Insulator	Both	Substrate [30]; Dielectric [30,31]
	Gelatin	Insulator	Both	Substrate [32,33]; Dielectric [34,35,36]
Synthetic Polymer	Poly(vinyl alcohol) (PVA)	Insulator	Biocompatible	Substrate [37,38]; Dielectric [39,40]
	Polydimethylsiloxane (PDMS)	Insulator	Biocompatible	Substrate [41]; Dielectric [42,43,44,45]
	Polylactide (PLA)	Insulator	Both	Substrate [46,47,48]; Dielectric [49]
	Polycaprolactone (PCL)	Insulator	Both	Dielectric [49]
	Poly(glycerol-co-sebacate) (PGS)	Insulator	Both	Dielectric [50]
	Poly(lactic-co-glycolic acid) (PLGA)	Insulator	Both	Substrate [51]; Dielectric [52]
	Polyaniline (PANI)	Conductor (doped)	Biocompatible	Conductor [53]
	Polypyrrole (PPy)	Conductor (doped)	Biocompatible	Conductor [54]
	Poly(3,4-ethylenedioxythiophene) (PEDOT)	Conductor (doped)	Biocompatible	Conductor [55]
